# Relapsing massive metal bezoar: a case report

**DOI:** 10.1186/1752-1947-3-56

**Published:** 2009-02-10

**Authors:** Manuel Rodrigo Prieto-Aldape, Francisco Issac Almaguer-García, Sandra Edith Figueroa-Jiménez, Oscar Fernández-Díaz, José Antonio Mora-Huerta, Alejandro González-Ojeda

**Affiliations:** 1 Surgical Division, Medical Research Unit, Clinical Epidemiology, Western National Medical Center, Mexican Institute of Social Security, Belisario Domínguez 1000, Guadalajara, Jalisco, Mexico; 2 Department of Surgery, Civil Hospital of Guadalajara "Fray Antonio Alcalde", Hospital 278, Guadalajara, Jalisco, Mexico

## Abstract

**Introduction:**

Bezoars are uncommon findings in the gastrointestinal tract and are composed of a wide variety of materials. We report a case of a relapsing metal bezoar in a man with schizophrenia.

**Case presentation:**

A 34-year-old man presented with a history of sub-acute onset of mild diffuse abdominal pain and abdominal distention. Physical examination revealed dullness to percussion in the upper and lower left quadrants. Past medical history was remarkable for epilepsy, schizophrenia and previous abdominal surgery for intestinal occlusion. Plain radiographs revealed objects of metal density contained within a dilated stomach. Celiotomy was performed revealing more than 350 metal objects inside the stomach. The patient was discharged and referred to a psychiatric facility.

**Conclusion:**

Intestinal occlusion in patients with psychiatric disorders can result from rare causes such as bezoars. This report alerts surgeons to rule out bezoars in the differential diagnosis of intestinal occlusion in people with mental health problems.

## Introduction

A bezoar is a conglomeration of partially digested or non-digested foreign material in the gastrointestinal (GI) tract, most commonly found in the stomach [1]. Less commonly, bezoars are found in the small intestine and the colon and only a few in the rectum are reported in the literature [2]. Bezoars may cause a wide variety of signs and symptoms depending on their location and can range from asymptomatic to occlusion and perforation. Bezoars are classified into several main types and are named according to the materials from which they are composed: phytobezoars, trichobezoars, pharmacobezoars and lactobezoars. Other rare, less frequent bezoars are unclassified and include materials such as plastic and metal.

The occurrence of relapse is rare and will reappear in 14% of cases, more often associated in psychiatric patients [3]. Only two cases of metal bezoar have been reported in the international literature [4,5]. To the best of our knowledge, we report the first case of a relapsing massive metal bezoar, managed at the Department of General Surgery of the "Hospital Civil de Guadalajara Fray Antonio Alcalde", University of Guadalajara.

## Case presentation

**Figure 1:**
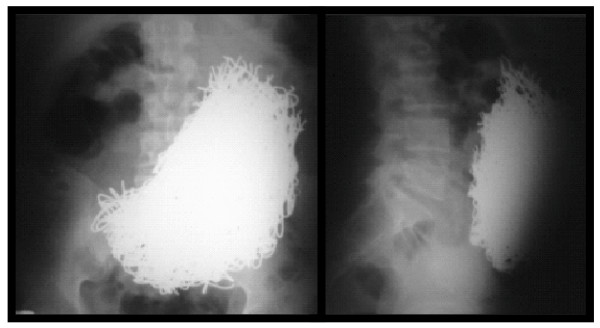
**Plain radiographs (anteroposterior and lateral) of the abdomen showing multiple objects of metal density contained within the stomach**.

A 34-year-old man presented to the emergency room with sub-acute onset of mild diffuse abdominal pain and abdominal distention, accompanied with vomiting of gastric and metal fragment contents. His social history was significant for tobacco and cocaine consumption. The patient was under treatment for long-term epilepsy and schizophrenia. He had an abdominal celiotomy two years earlier for removal of foreign metal contents in the stomach. Physical examination revealed an afebrile patient with tachycardia and tachypnea. The abdomen was distended and tender to palpation without peritoneal signs of irritation. A palpable mass was felt in the upper and lower left quadrants both being dull to percussion. Liver dullness was not obliterated and rectal examination revealed an empty rectum. Laboratory tests revealed leukocytosis with left shift, other tests (liver function tests, serum electrolytes and blood gases) were unremarkable. Plain radiographs (anteroposterior (AP) and lateral) of the abdomen showed multiple objects of metal density contained within the stomach (Figure 1).

**Figure 2:**
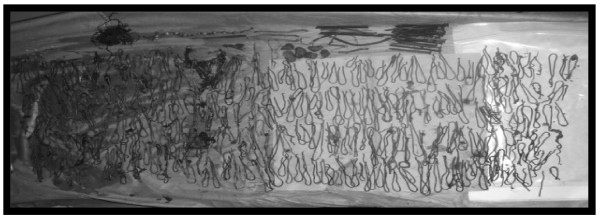
**Objects obtained from the stomach**.

The patient was taken to the operating room (OR) for an exploratory celiotomy through a midline incision. He was found to have a grossly dilated stomach. A longitudinal gastrostomy was made revealing multiple metal objects including: nails, copper wires, stones, plastic rosary beads and the remains of partially digested food (389 objects) (Figure 2). After emptying the stomach, it was closed with a double layer suture in a custom fashion leaving in place a nasogastric tube for gastric drainage. Before closing the abdomen, a complete exploration of the small and large bowel was made to avoid remnants of metal particles to prevent any postoperative complication. Postoperative recovery was uneventful. The patient was discharged and transferred to a psychiatric facility 8 days after surgery. A 6-month follow-up showed no recurrence or any postoperative complication.

## Discussion

Bezoars are classified according to the materials which they are composed of (named in order of frequency): *Phytobezoar *made of vegetable fibers or plant material, *trichobezoar *are a result of ingestion of human hair, *drug-bezoar *contain accumulated masses of medication, *lactobezoars *made of undigested milk described in premature infants and in full term infants and other less frequent materials are named miscellaneous bezoars or *polybezoars *[6]. Gastrointestinal bezoar is uncommon and is reported to occur in 4% of all admissions for small-bowel obstruction [7]. Reports of bezoars causing obstruction of the gastrointestinal tract have existed since the late 18th century. Around 400 cases of trichobezoar and a larger number of phytobezoars have been reported in the literature [8]. After reviewing the literature, we found only two previous reports of "metal bezoar", the first in 1956 by Salb [4] and the second reported by Kaplan *et al. *[5] in 2005, none being relapsing or massive. Approximately 10% of patients show psychiatric abnormalities or mental retardation [8] therefore psychiatric evaluation and therapy are needed to prevent a recurrence [9]. As in our patient, it is obvious that inadequate psychiatric follow-up can lead to a life-threatening recurrence.

This entity occurs in normal stomachs caused by the ingestion of materials that cannot pass the pylorus such as plastic, metal and wooden foreign bodies. Other bezoars occur as a complication of gastric motility, usually prior gastric surgery such as vagotomy, leading to reduced gastric acidity; gastric stasis and loss of pyloric function; peptic ulcer disease/stenosis; chronic gastritis; Crohn's disease; carcinoma of the stomach, duodenum, or pancreas; dehydration and hypothyroidism [10]; diabetic patients with neuropathy or myotonic dystrophy [11]. Also to be considered is the fact that certain medications that decrease GI motility, such as anticholinergic agents, ganglionic blocking agents, and opiates, may also give rise to a bezoar [10]. With time, undigested foreign bodies are retained by mucus and become enmeshed, creating a mass in the shape of the stomach where they are usually found. They may attain large sizes owing to the chronicity of the problem and delayed reporting by the patients [12].

Bezoars have been known to cause a wide variety of symptoms. In the stomach, they are associated with anorexia, bloating, early satiety, dyspepsia, malaise, weakness, weight loss, headaches, and a feeling of fullness or heaviness in the epigastrium [13]. They may also present with gastrointestinal bleeding (6%) and intestinal obstruction or perforation (10%) [14]. Gastrointestinal bezoars can be easily diagnosed in most patients. Plain X-rays, like the radiograph in our patient, are unique and lead to the diagnosis, however diagnostic difficulties arise in patients with radiolucent bezoars, and contrast studies of the GI tract by radiography and computed tomography (CT) scan are necessary in such circumstances. Upper GI endoscopy is the method of choice in detecting esophageal, gastric and duodenal foreign bodies. Occasionally, bezoars are found incidentally when an emergency laparotomy is done secondarily to bowel obstruction. Several treatments have been proposed for bezoars and they depend upon the clinical presentation as well as on the composition of the bezoar. Chemical and enzymatic compounds have been used for dissolution of esophageal and gastric phytobezoars and lactobezoars [15]. For small bezoars, endoscopy has been the treatment of choice. Once the obstruction occurs, surgery is the only way to solve the problem. Frequently, synchronous bezoars are found in the stomach or other areas of the gastrointestinal tract; therefore it is mandatory to carry out a thorough exploration of the small intestine and colon [11] to avoid recurrence of intestinal obstruction due to a retained bezoar. After discharge, recurrence has been reported in up to 14% of cases, especially in patients with psychiatric disturbances and with previous gastric surgery [3].

## Conclusion

In our patient, the diagnosis was simple because there were not many options; a patient with intestinal obstruction, psychiatric disturbances, previous gastric surgery and the radiographic findings took him immediately to the OR. The gastrostomy with foreign material removal and thorough exploration of the rest of the GI tract was curative. We conclude that, in the face of an intestinal occlusion in a patient with a psychiatric disorder and a history of GI surgery, the physician must suspect, in addition to adhesions or hernias, the possibility of a bezoar.

Plain and contrast enhanced radiographic studies are important for both diagnosis and treatment. It is also important to consider GI endoscopy as part of the diagnosis and treatment plan. In cases where bezoars cannot be treated by dissolving agents such as enzymes or chemicals and endoscopy is not able to remove the obstruction, surgery is the last option.

## Consent

Written informed consent was obtained from the patient's mother for publication of this case report and any accompanying images. A copy of the written consent is available for review by the Editor-in-Chief of this journal.

## Competing interests

The authors declare that they have no competing interests.

## Authors' contributions

MRPA participated in the surgical team and the care of the patient during his hospitalization, and was also involved in drafting and review of the manuscript. FIAG made the evaluation in the emergency department, the diagnostic and treatment protocol, and the postoperative care, and also the review of the literature. SEFJ participated in the surgical team and the care of the patient, and also took the pictures, carried out the review of the literature and participated in the drafting of the manuscript. OFD and JAMH performed the case review, completed the documentation and were major contributors in writing the manuscript. AGO reviewed the case, evaluated the available literature, and made a substantive intellectual contribution to the final version. All authors read and approved the final manuscript.
